# Draft Genome Sequence of *Acinetobacter bereziniae* HPC229, a Carbapenem-Resistant Clinical Strain from Argentina Harboring *bla*_NDM-1_

**DOI:** 10.1128/genomeA.00117-16

**Published:** 2016-03-10

**Authors:** Marco Brovedan, Patricia M. Marchiaro, Jorgelina Morán-Barrio, Santiago Revale, Marcela Cameranesi, Luciano Brambilla, Alejandro M. Viale, Adriana S. Limansky

**Affiliations:** a Instituto de Biología Molecular y Celular de Rosario (IBR, CONICET), Facultad de Ciencias Bioquímicas y Farmacéuticas, Universidad Nacional de Rosario, Rosario, Argentina; b Instituto de Agrobiotecnología Rosario (INDEAR), Rosario, Argentina

## Abstract

We report here the draft genome sequence of an NDM-1-producing *Acinetobacter bereziniae* clinical strain, HPC229. This strain harbors both plasmid and chromosomal resistance determinants toward different β-lactams and aminoglycosides as well as several types of multidrug efflux pumps, most likely representing an adaptation strategy for survival under different environments.

## GENOME ANNOUNCEMENT

*Acinetobacter bereziniae* is isolated primarily from clinical specimens and health care-associated environments (1). Although most *A. bereziniae* isolates are susceptible to antimicrobials (2), clinical strains bearing metallo-beta-lactamase genes have just been reported (3-6). We recently described the sequence of a *bla*_NDM-1_-harboring plasmid (pNDM229, KT072713.1) carried by a carbapenem-resistant *A. bereziniae* strain (HPC229) from Argentina (4). We report here the whole-genome sequencing (WGS) of HPC229, which constitutes the sixth *A. bereziniae* genome in databases. Genes involved in β-lactam and aminoglycoside resistance were found in the HPC229 genome. Moreover, genes involved in different efflux pump systems associated with resistance to antimicrobials and toxic compounds were identified (7, 8). These findings support the notion that *A. bereziniae* represents an environmental reservoir of resistance genes of clinical relevance.

HPC229 DNA was prepared using the Wizard genomic DNA purification kit (Promega) and subjected to 454 pyrosequencing (Roche Diagnostics Corporation) at INDEAR, Rosario. Data generated were assembled using Newbler v2.9, resulting in 134 contigs, 5 of them constituting the pNDM229 plasmid (4). The remaining 129 contigs have a length of 4,596,631 bp and a G+C content of 38.00%. Genome annotation was done using the NCBI Prokaryotic Genomes Annotation Pipeline (9), and eight contigs were left out for being shorter than 200 bp. The RAST server was employed for subsystem classification and functional annotation (10) and then the genome was manually curated. Complementary gene identification analyses were done using Mauve (11), ISFinder (12), Res-Finder 2.1 (13), and TCDB (14). A total of 4,124 protein-coding sequences, 4 rRNAs, and 62 tRNA genes were predicted by these analyses.

WGS analyses revealed genes associated with β-lactam resistance, including *bla*_NDM-1_ (4), *ampC*, a new *bla*_OXA-229_-like (15) variant, and 3 other putative β-lactamase genes, as well as a new *carO* allele coding for an outer membrane protein associated with imipenem uptake (16). Other resistance genes included *aphA6* (4), and a putative phosphotransferase encoding resistance to aminoglycosides. Efflux pump- and membrane-associated transporter genes of different superfamilies included (7, 8, 14, 17) ABC (MacA, MacB); BART (Acr3); IT (ArsB); MOP (NorM, AbeM); MFS (CraA, SmvA, MFS transporter, Bcr/CflA, MFS permease); DMT (AbeS, QaceΔ1-like); MER (MerT, MerC); and RND (AdeABC, AdeIJK, AdeE, CzcABC, and CusABC). The latter operon, absent in the other *A. bereziniae* genomes, exhibits 95% nucleotide identity to a portion of the GI2 genomic island in *A. baumannii* LAC-4 (8). Downstream from *cusABC*, there are regions encompassing IS*Aba2*, *acr3*, and *feoAB* genes, with *acr3* showing the highest identity to its ortholog of *A. tandoii* DSM14970, whereas *feoAB* was identified in the *A. baumannii* LAC-4 GI2 immediately downstream of its *cusABC* operon. These observations suggest a chimeric construct in HPC229 derived from gene exchange among *Acinetobacter* species. WGS analyses will provide further evidence of the ability of *A. bereziniae* to act as a reservoir of resistance genes and may help to understand the adaptability mechanisms of *Acinetobacter* in response to environmental challenges.

### Nucleotide sequence accession numbers.

This WGS project has been deposited at DDBJ/EMBL/GenBank under the accession LKDJ00000000. We describe here the version LKDJ00000000.1.

## References

[1] Nemec A, Musílek M, Sedo O, De Baere T, Maixnerová M, van der Reijden TJ, Zdráhal Z, Vaneechoutte M, Dijkshoorn L 2010 *Acinetobacter bereziniae* sp. nov. and *Acinetobacter guillouiae* sp. nov., to accommodate *Acinetobacter* genomic species 10 and 11, respectively. Int J Syst Evol Microbiol 60:896–903. doi:10.1099/ijs.0.013656-0. 19661501

[2] Zander E, Seifert H, Higgins PG 2014 Insertion sequence IS18 mediates overexpression of *bla*_OXA-257_ in a carbapenem-resistant *Acinetobacter bereziniae* isolate. J Antimicrob Chemother 69:270–272. doi:10.1093/jac/dkt313. 23934740

[3] Lee K, Kim CK, Hong SG, Choi J, Song S, Koh E, Yong D, Jeong SH, Yum JH, Docquier JD, Rossolini GM, Chong Y 2010 Characteristics of clinical isolates of *Acinetobacter* genomospecies 10 carrying two different metallo-beta-lactamases. Int J Antimicrob Agents 36:259–263. doi:10.1016/j.ijantimicag.2010.05.018. 20599361

[4] Brovedan M, Marchiaro PM, Morán-Barrio J, Cameranesi M, Cera G, Rinaudo M, Viale AM, Limansky AS 2015 Complete sequence of a *bla*_NDM-1_-harboring plasmid in an *Acinetobacter bereziniae* clinical strain isolated in Argentina. Antimicrob Agents Chemother 59:6667–6669. doi:10.1128/AAC.00367-15. 26248354 PMC4576080

[5] Chagas TPG, Carvalho-Assef APDA, Martins Aires CA, Bertocini R, Asensi MD 2015 Detection of an NDM-1-producing *Acinetobacter bereziniae* strain in Brazil. J Glob Antimicrob Resist 3:147–148. doi:10.1016/j.jgar.2015.03.005. 27873666

[6] Jones LS, Carvalho MJ, Toleman MA, White PL, Connor TR, Mushtaq A, Weeks JL, Kumarasamy KK, Raven KE, Török ME, Peacock SJ, Howe RA, Walsh TR 2015 Characterization of plasmids in extensively drug-resistant *Acinetobacter* strains isolated in India and Pakistan. Antimicrob Agents Chemother 59:923–929. doi:10.1128/AAC.03242-14. 25421466 PMC4335910

[7] Li X-Z, Plésiat P, Nikaido H 2015 The challenge of efflux-mediated antibiotic resistance in gram-negative bacteria. Clin Microbiol Rev 28:337–418. 25788514 10.1128/CMR.00117-14 PMC4402952

[8] Ou HY, Kuang SN, He X, Molgora BM, Ewing PJ, Deng Z, Osby M, Chen W, Xu HH 2015 Complete genome sequence of hypervirulent and outbreak-associated *Acinetobacter baumannii* strain LAC-4: epidemiology, resistance genetic determinants and potential virulence factors. Sci Rep 5:8643. doi:10.1038/srep08643. 25728466 PMC4345345

[9] Angiuoli SV, Gussman A, Klimke W, Cochrane G, Field D, Garrity G, Kodira CD, Kyrpides N, Madupu R, Markowitz V, Tatusova T, Thomson N, White O 2008 Toward an online repository of standard operating procedures (SOPs) for (meta)genomic annotation. Omics 12:137–141. doi:10.1089/omi.2008.0017. 18416670 PMC3196215

[10] Aziz RK, Bartels D, Best AA, DeJongh M, Disz T, Edwards RA, Formsma K, Gerdes S, Glass EM, Kubal M, Meyer F, Olsen GJ, Olson R, Osterman AL, Overbeek RA, McNeil LK, Paarmann D, Paczian T, Parrello B, Pusch GD, Reich C, Stevens R, Vassieva O, Vonstein V, Wilke A, Zagnitko O 2008 The RAST Server: rapid annotations using subsystems technology. BMC Genomics 9:75. doi:10.1186/1471-2164-9-75. 18261238 PMC2265698

[11] Darling AC, Mau B, Blattner FR, Perna NT 2004 Mauve: multiple alignment of conserved genomic sequence with rearrangements. Genome Res 14:1394–1403. doi:10.1101/gr.2289704. 15231754 PMC442156

[12] Siguier P, Perochon J, Lestrade L, Mahillon J, Chandler M 2006 ISfinder: the reference centre for bacterial insertion sequences. Nucleic Acids Res 34:D32–D36. doi:10.1093/nar/gkj014. 16381877 PMC1347377

[13] Zankari E, Hasman H, Cosentino S, Vestergaard M, Rasmussen S, Lund O, Aarestrup FM, Larsen MV 2012 Identification of acquired antimicrobial resistance genes. J Antimicrob Chemother 67:2640–2644. doi:10.1093/jac/dks261. 22782487 PMC3468078

[14] Saier MH, Reddy VS, Tamang DG, Västermark A 2014 The transporter classification database. Nucleic Acids Res 42:D251–D258. doi:10.1093/nar/gkt1097. 24225317 PMC3964967

[15] Bonnin RA, Ocampo-Sosa AA, Poirel L, Guet-Revillet H, Nordmann P 2012 Biochemical and genetic characterization of carbapenem-hydrolyzing β-lactamase OXA-229 from *Acinetobacter bereziniae*. Antimicrob Agents Chemother 56:3923–3927. doi:10.1128/AAC.00257-12. 22508298 PMC3393453

[16] Mussi MA, Limansky AS, Relling V, Ravasi P, Arakaki A, Actis LA, Viale AM 2011 Horizontal gene transfer and assortative recombination within the *Acinetobacter baumannii* clinical population provide genetic diversity at the single *carO* gene, encoding a major outer membrane protein channel. J Bacteriol 193:4736–4748. doi:10.1128/JB.01533-10. 21764928 PMC3165691

[17] Chau S-L, Chu Y-W, Houang ETS 2004 Novel resistance-nodulation-cell division efflux system AdeDE in *Acinetobacter* genomic DNA group 3. Antimicrob Agents Chemother 48:4054–4055. doi:10.1128/AAC.48.10.4054-4055.2004. 15388479 PMC521926

